# Antibodies Against Hepatitis E Virus (HEV) in European Moose and White-Tailed Deer in Finland

**DOI:** 10.1007/s12560-020-09442-0

**Published:** 2020-09-07

**Authors:** Emil Loikkanen, Satu Oristo, Natalia Hämäläinen, Pikka Jokelainen, Tuija Kantala, Antti Sukura, Leena Maunula

**Affiliations:** 1 Department of Food Hygiene and Environmental Health, Faculty of Veterinary Medicine, University of Helsinki, Helsinki, Finland; 2 Infectious Disease Preparedness, Statens Serum Institut, Copenhagen, Denmark; 3 Department of Veterinary Biosciences, Faculty of Veterinary Medicine, University of Helsinki, Helsinki, Finland; 4 Virology Unit, Finnish Food Authority, Helsinki, Finland

**Keywords:** Hepatitis E virus, HEV, Seroprevalence, Cervid, Zoonosis

## Abstract

The main animal reservoirs of zoonotic hepatitis E virus (HEV) are domestic pigs and wild boars, but HEV also infects cervids. In this study, we estimated the prevalence of HEV in Finnish cervid species that are commonly hunted for human consumption. We investigated sera from 342 European moose (*Alces alces*), 70 white-tailed deer (*Odocoileus virginianus*), and 12 European roe deer (*Capreolus capreolus*). The samples had been collected from legally hunted animals from different districts of Finland during 2008–2009. We analysed the samples for total anti-HEV antibodies using a double-sandwich ELISA assay. Seropositive sera were analysed with RT-qPCR for HEV RNA. HEV seroprevalence was 9.1% (31/342) in moose and 1.4% (1/70) in white-tailed deer. None of the European roe deer were HEV seropositive (0/12). No HEV RNA was detected from samples of seropositive animals. HEV seropositive moose were detected in all districts. Statistically, HEV seroprevalence in moose was significantly higher (*p* < 0.05) in the North-East area compared to the South-West area. The highest HEV seroprevalence (20.0%) in district level was more than six times higher than the lowest (3.1%). We demonstrated the presence of total anti-HEV antibodies in European moose and white-tailed deer in Finland. Our results suggest that HEV is circulating among the moose population. Infections may occur also in white-tailed deer. We were the first to report a HEV seropositive white-tailed deer from Europe. Further studies are needed to demonstrate the HEV genotypes in cervids in Finland and to evaluate the importance of the findings in relation to food safety.

## Introduction

Hepatitis E virus (HEV) is a positive-sense, single-stranded RNA virus that is classified in the family of *Hepeviridae*, genus of *Orthohepevirus*, and species of *Orthohepevirus A* (Smith et al. 2014). Currently, eight genotypes (HEV-1–HEV-8) are recognised and five of them can infect humans (Smith et al. 2014; Sridhar et al. 2017). HEV-1 and HEV-2 are human specific and endemic mainly in tropical and subtropical regions (Forni et al. 2018). HEV-3, HEV-4, and HEV-7 are zoonotic (Sridhar et al. 2017). HEV-3 occurs worldwide (Forni et al. 2018), and it is the most important zoonotic genotype in Europe (EFSA 2017). HEV-4 is mostly restricted to Asia and Europe, while HEV-7 has been found in dromedaries in the Greater Middle East (Forni et al. 2018). The main animal reservoirs of HEV-3 and HEV-4 are domestic pigs and wild boars, but both genotypes can also infect numerous other animals, including cervids (Kenney 2019).

Unlike human-specific HEV, which is linked to large epidemics and high mortality in pregnant women (Patra et al. 2007), zoonotic HEV usually causes sporadic cases of asymptomatic infection or acute hepatitis in humans (Kantala and Maunula 2018). Zoonotic HEV infection can also cause neurological symptoms, chronic liver disease, and even death, especially in immunosuppressed humans (Kantala and Maunula 2018). The number of HEV, especially HEV-3, cases in humans has increased in the last decade and most have been linked to food products (EFSA 2017). In general, transmission of zoonotic HEV occurs through consumption of raw or undercooked food originating from infected animals, but also through direct contact with infected animals or contaminated environment (Yugo and Meng 2013).

In animals, zoonotic HEV is usually subclinical (Yugo and Meng 2013). Studies of HEV in cervids have mostly focused on European roe deer (*Capreolus capreolus*), red deer (*Cervus elaphus*), and sika deer (*Cervus nippon*) but fallow deer (*Dama dama*), white-tailed deer (*Odocoileus virginianus*), and European moose (*Alces alces*) have also been studied (Yu et al. 2007; Zhang et al. 2008; Reuter et al. 2009; Boadella et al. 2010; Forgách et al. 2010; Rutjes et al. 2010; Dong et al. 2011; Medrano et al. 2012; Lin et al. 2014, 2015; Larska et al. 2015; Lhomme et al. 2015; Serracca et al. 2015; Kubankova et al. 2015; Zhang et al. 2015; Kukielka et al. 2016; Neumann et al. 2016; Roth et al. 2016; Anheyer-Behmenburg et al. 2017; Di Bartolo et al. 2017; Thiry et al. 2017; Weger et al. 2017; Spancerniene et al. 2018; Trojnar et al. 2020).

In the species of the deer family (from here on referred to as “deer”), except European moose, the reported HEV prevalences vary based on the species and the country. Both HEV-3 and HEV-4 have been detected in deer and have caused human infections via raw venison (Tei et al. 2003; Takahashi et al. 2004; Choi et al. 2013). However, only HEV-3 has been found in deer in Europe (Reuter et al. 2009; Boadella et al. 2010; Forgách et al. 2010; Serracca et al. 2015; Kubankova et al. 2015; Kukielka et al. 2016; Anheyer-Behmenburg et al. 2017; Di Bartolo et al. 2017; Thiry et al. 2017; Spancerniene et al. 2018). HEV seroprevalences have ranged 0.0–13.9% in Europe, and related HEV RNA prevalences have ranged 0.0–34.4% (Reuter et al. 2009; Boadella et al. 2010; Forgách et al. 2010; Rutjes et al. 2010; Larska et al. 2015; Lhomme et al. 2015; Serracca et al. 2015; Kubankova et al. 2015; Kukielka et al. 2016; Neumann et al. 2016; Roth et al. 2016; Anheyer-Behmenburg et al. 2017; Di Bartolo et al. 2017; Thiry et al. 2017; Spancerniene et al. 2018; Trojnar et al. 2020). HEV RNA prevalences over 10% have been reported in Hungary (Reuter et al. 2009; Forgárch et al. 2010), Lithuania (Spancerniene et al. 2018), Italy (Di Bartolo et al. 2017), and Spain (Boadella et al. 2010; Kukielka et al. 2016). In Italy and Spain, HEV seroprevalences were also over 10% (Boadella et al. 2010; Kukielka et al. 2016; Di Bartolo et al. 2017). Globally, the highest HEV seroprevalence, 62.7%, was recorded in Mexico from ranched white-tailed deer (Medrano et al. 2012).

In European moose (from here on referred to as “moose”), HEV has been studied from larger sample numbers previously only in Sweden, where HEV seroprevalence was 18.6% and HEV RNA prevalence was 14.7% (Lin et al. 2015), and Lithuania, where HEV RNA prevalence was 7.7% (Spancerniene et al. 2018). The HEV genotype detected in moose in Sweden is potentially new and has unknown zoonotic potential (Lin et al. 2014).

In Finland, the prevalence of HEV has been mostly studied in humans and domestic pigs, and anti-HEV antibodies and HEV RNA have been found in both (Kantala et al. 2009, 2013, 2015, 2017). All human HEV infections were caused by HEV-1 in a study using sera collected from hepatitis patients in 2000–2008 in Finland (Kantala et al. 2009). More recently, the first report of an autochthonous case of severe acute hepatitis caused by HEV-3 has been documented (Kettunen et al. 2013). Only HEV-3 has been found from pigs in Finland, with varying prevalence of 0.0–47.6% depending on the age of the pigs (Kantala et al. 2013, 2015). HEV seroprevalence in pigs at the time of slaughter was 84.0% (Kantala et al. 2013).

No similar studies have focused on HEV in cervids in Finland. According to the Natural Resources Institute Finland (Luke 2019a), over 120,000 cervids were hunted in Finland in 2018. Hunters handle the carcasses by themselves in the forest and in local facilities (Laaksonen and Paulsen 2015; Schielke et al. 2015). In the study of Chaussade et al. (2013), hunting was a risk factor for HEV seropositivity. However, in the study of Ivanova et al. (2015), HEV seroprevalence in hunters was significantly lower than in pig farm workers who are also at risk for HEV infections. Estimated consumption of cervid meat was 1.8 kg per person in Finland in 2017 (Luke 2019b). Based on this information, it is important to know if people are exposed to HEV by handling cervid carcasses or eating meat from hunted cervids in Finland.

We sought to estimate the HEV seroprevalence in cervids in Finland. This first estimate would serve as a needed baseline for HEV seroprevalence. Furthermore, we intended to screen the possible seropositive samples for HEV RNA to detect acute infections. We also wanted to evaluate whether the cervid species, cervid density, or geographical region affect the prevalence. We hypothesised that HEV infections would occur in cervids in Finland as they occur in the neighbouring country, Sweden.

## Materials and Methods

### Sample Material

Our sampling frame was the collection of cervid sera stored frozen at the Department of Veterinary Biosciences, the Faculty of Veterinary Medicine, University of Helsinki. The samples were originally collected from wild, free-ranging cervids during the hunting season of 2008–2009 for a seroepidemiological study on *Toxoplasma gondii* (Jokelainen et al. 2010). Background information, including species, age group (calf, adult), sex, and hunting district where the animal was hunted, were received with the blood samples. All of the animals were legally hunted for human consumption.

**Fig. 1:**
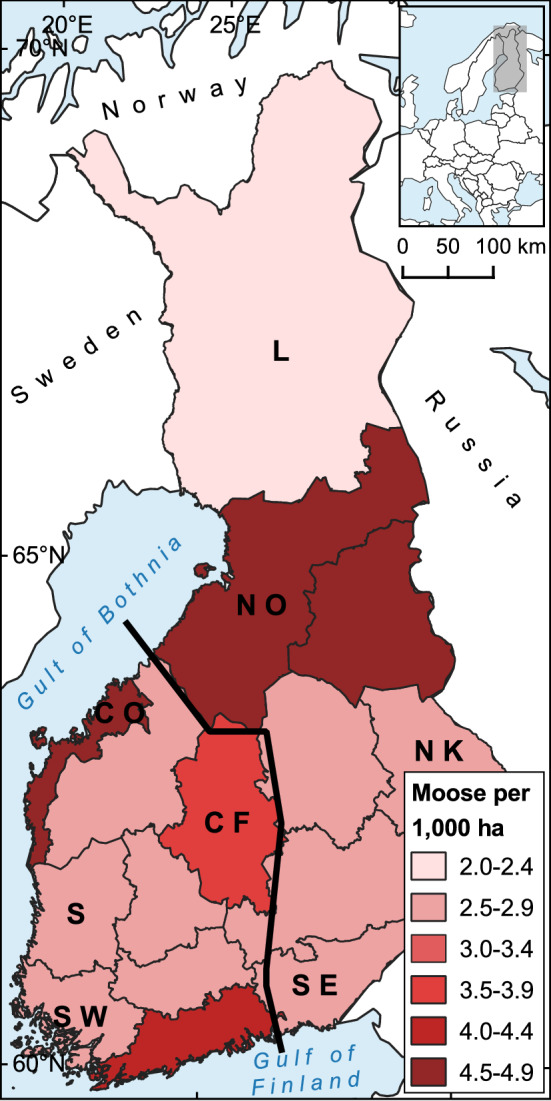
Map of European moose densities in Finnish game management districts including studied districts. Moose densities are presented as number of moose per 1000 ha (Riista-ja kalatalouden tutkimuslaitos 2009). The game management districts included in this study are labelled with abbreviations. The line divides the districts into the North-East and South-West areas. *L* Lapland, *NO* North Ostrobothnia, *CO* Coastal Ostrobothnia, *CF* Central Finland, *NK* North Karelia, *S* Satakunta, *SW* Southwest Finland, and *SE* Southeast Finland

We included 342 moose sera from seven game management districts in this study (Table 1, Fig. 1). The districts were selected from different parts of Finland and had different moose densities (Fig. 1). They were grouped as North-East (Lapland, Northern Ostrobothnia, Northern Karelia, and Southeast Finland; 194 serum samples) and South-West (Coastal Ostrobothnia, Central Finland, and Southwest Finland; 148 serum samples) areas (Fig. 1). As for the deer samples, we included 70 white-tailed deer sera and 12 European roe deer sera (Table 1). These samples originated from two game management districts in South-West (Satakunta and Southwest Finland; Fig. 1) where these deer species are most numerous (Jokelainen et al. 2010). Total number of samples included was based on availability and the same number of samples were chosen from the two age groups except for European roe deer due to limited number of samples from calves. The samples from each district were randomly selected using a random number generator.

**Table 1:** Cervid samples included in the study according to the species, hunting districts, age groups, and sex, as well as moose densities in the studied hunting districts

Species	District	Number of samples	Age group		Sex			Moose density (number/1000 ha^a^)
			Adult (%)	Calf (%)	Female (%)	Male (%)	Unknown (%)	
European moose	Lapland, L	32	16 (50.0)	16 (50.0)	18 (56.3)	14 (43.8)	–	2.1
	North Ostrobothnia, NO	50	25 (50.0)	25 (50.0)	22 (44.0)	28 (56.0)	–	4.5
	Coastal Ostrobothnia, CO	34	17 (50.0)	17 (50.0)	16 (47.1)	17 (50.0)	1 (2.9)	4.7
	Central Finland, CF	50	25 (50.0)	25 (50.0)	25 (50.0)	25 (50.0)	–	3.5
	North Karelia, NK	46	23 (50.0)	23 (50.0)	13 (28.3)	33 (71.7)	–	2.5
	Southwest Finland, SW	64	32 (50.0)	32 (50.0)	32 (50.0)	32 (50.0)	–	2.7
	Southeast Finland, SE	66	33 (50.0)	33 (50.0)	24 (36.4)	42 (63.6)	–	2.9
	Total	342	171 (50.0)	171 (50.0)	150 (43.9)	191 (55.8)	1 (0.3)	3.2
White-tailed deer	Satakunta, S	20	10 (50.0)	10 (50.0)	7 (35.0)	13 (65.0)	–	–
	Southwest Finland, SW	50	25 (50.0)	25 (50.0)	19 (38.0)	30 (60.0)	1 (2.0)	–
	Total	70	35 (50.0)	35 (50.0)	26 (37,1)	43 (61.4)	1 (1.4)	–
European roe deer	Satakunta, S	6	5 (83.3)	1 (16.7)	2 (33.3)	4 (66.7)	–	–
	Southwest Finland, SW	6	4 (66.7)	2 (33.3)	2 (33.3)	4 (66.7)	–	–
	Total	12	9 (75.0)	3 (25.0)	4 (33.3)	8 (66.7)	–	–

^a^Estimated number of moose per 1000 ha (Riista-ja kalatalouden tutkimuslaitos 2009)

### Detection of Total Anti-HEV Antibodies by ELISA

For the detection of anti-HEV antibodies, we analysed the sera with a commercial HEV-Ab ELISA kit (Axiom Diagnostic, Germany) as previously described (Kantala et al. 2013). The HEV-Ab ELISA kit is a double-antigen sandwich ELISA assay for qualitative detection of total anti-HEV antibodies (IgM, IgG, etc.) and is suitable for testing animal sera. The samples were tested in duplicates. Washing of the ELISA plate was performed manually. Reading of the results was repeated three times by using Thermo Scientific Multiskan FC (Thermo Fisher Scientific, Finland) with a 450 nm filter. Calculations were made according to the kit’s instructions. Only samples with at least one truly positive result (the individual absorbance of sample/cut-off value > 1.1) were considered as positive in our study.

### Detection of HEV RNA by RT-qPCR

We used quantitative reverse transcription polymerase chain reaction (RT-qPCR) to screen for HEV RNA from the HEV seropositive animals’ sera to detect possible acute HEV infections. From each HEV seropositive animals’ sample, 140 µl of undiluted sera was analysed. Before RNA extraction, we added 10 µl of whole mengovirus (strain MC0 grown in HeLa cells; kindly donated from Bosch A, University of Barcelona, Spain) to the samples to control the RNA extraction efficiency. RNA extraction was done using a commercial kit (E.Z.N.A.® Viral RNA Kit, Omega Bio-tek, United States) according to the manufacturer's instructions without using Carrier RNA and with an elution volume of 70 µl. We cleaned the extracted RNA samples with a commercial inhibitor removal kit (OneStep™ PCR Inhibitor Removal, Zymo Research, United States) following the manufacturer's instructions. In the presence of inhibition the sample was diluted.

To measure the presence of HEV RNA, we used the method described earlier (Kantala et al. 2013) with slight modifications. Briefly, we used QuantiTect Probe RT-PCR kit (Qiagen, German) for real-time one-step RT-PCR: the 20 μl reaction volume included 10 μl of 2 × QuantiTect Probe RT-PCR Master Mix, 0.2 μl of QuantiTect RT Mix, 0.9 μM of primers, 0.3 μM of probe, and 5 μl of the sample. We used primers and a probe according to Jothikumar et al. (2006). We performed RT-PCR with a Rotor-Gene 3000 Instrument (Corbett Life Sciences, Sydney, Australia) and HEV Probe RT-PCR running programme with the following steps: 50 °C 30 min; 95 °C 15 min; and 50 cycles of 95 °C 15 s, 55 °C 45 s, 72 °C 45 s. For mengovirus, the same RT-PCR programme was used with the primers and probe according to ISO 15,216–1:2017 (2017) and a 20 μl reaction volume containing 10 μl of 2 × QuantiTect Probe RT-PCR Master Mix, 0.2 μl of QuantiTect RT Mix, 1 μM of primers, 0.2 μM of probe, and 5 μl of the sample. We controlled for PCR inhibitors with EC RNA of human norovirus (HuNV) GI (Ballesté et al. 2020, https://aquavalens.org/project/latest-results-cluster-1). HuNV RNA was detected using the methods of ISO 15,216–1:2017 (2017): the 21 µl reaction volume included 10 μl of 2 × QuantiTect Probe RT-PCR Master Mix, 0.2 μl of QuantiTect RT Mix, 0.9 μM of primers, 0.3 μM of probe, 5 μl of the sample, and 1 μl of the EC HuNV RNA. For HuNV RT-PCR, we ran a programme with the following steps: 53 °C 30 min; 95 °C 15 min; and 45 cycles of 95 °C 15 s, 58 °C 45 s, 72 °C 45 s. We considered the RNA extraction to be successful if the difference between control and sample cycle thresholds was a maximum of six cycles for the mengovirus RT-PCR. PCR inhibitors were deemed to be at tolerated level if the difference was a maximum of two cycles in the HuNV RT-PCR.

### Statistical Methods

We evaluated the sample sizes using Epitools Epidemiological Calculators (https://epitools.ausvet.com.au/). We based the calculations on HEV seroprevalence in moose and European roe deer in Sweden (Lin et al. 2015; Roth et al. 2016) and in white-tailed deer in Canada (Weger et al. 2017). Desired precision in the calculations was ± 5.0%, and population sizes were infinite.

We calculated the confidence intervals (CI) for the seroprevalence estimates by Wilson score interval of 95%, evaluated differences (*p* < 0.05 considered significant) by Fisher’s Exact Test, and effect sizes by *φ* test. Correlation between moose densities and HEV seroprevalence in moose was calculated with Pearson Correlation. All statistical analyses were performed using the IBM SPSS Statistics 25 programme (IBM).

## Results

### Total Anti-HEV Antibodies in Moose

**Fig. 2:**
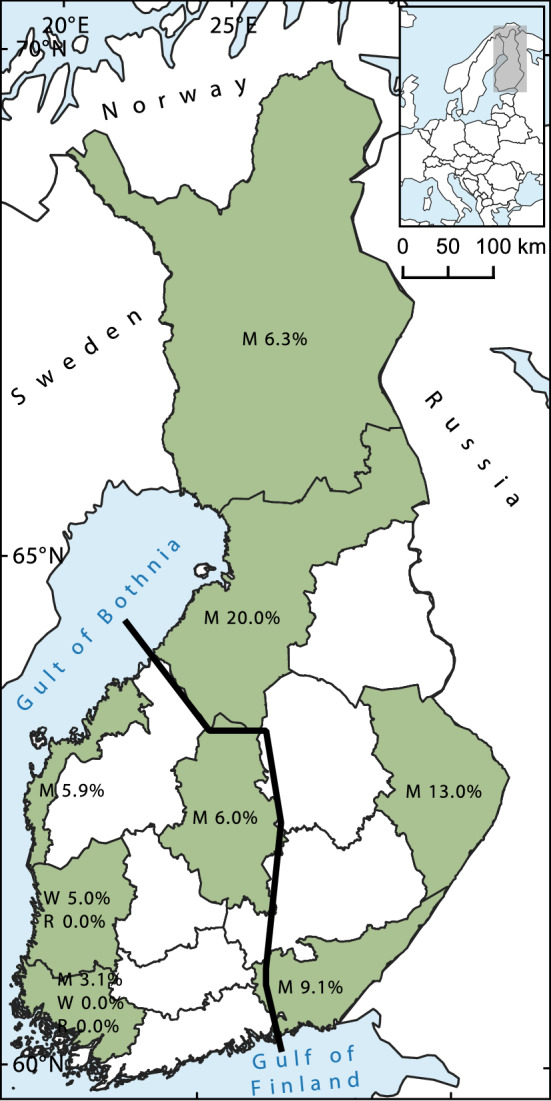
Map of observed HEV seroprevalences in different cervid species in Finland. HEV seroprevalences (%) observed in European moose (M), white-tailed deer (W), and European roe deer (R) by the game management districts. The line divides the districts into the North-East and South-West areas

We detected 31 of the 342 moose to be HEV seropositive (9.1%, CI 6.5–12.6%; Table 2). Positive samples were found from all studied districts (Fig. 2). Significantly higher HEV seroprevalence was detected in the North-East area (12.4%, CI 8.5–17.7%) compared to the South-West area (4.7%, CI 2.3–9.4%; *p* = 0.021, *φ* = − 0.132). There were no statistically significant differences in the seroprevalences between the individual districts when all districts were included in the evaluation. However, when data from two districts at a time were included, we detected a statistically significant difference (*p* = 0.005, *φ* = − 0.273) in seroprevalence between Northern Ostrobothnia and Southwest Finland which had the highest (20.0%) and the lowest (3.1%) seroprevalence, respectively (Table 2). No correlation between moose density and HEV seroprevalence was detected. There were no significant differences in seroprevalences depending on the age group or the sex of the moose.

**Table 2:** Total anti-HEV antibody prevalence in European moose in Finland

Variable	Number of samples (positive/total)	HEV seroprevalence % (95% CI)
District		
Lapland, L	2/32	6.3 (1.7–20.1)
North Ostrobothnia, NO	10/50	20.0 (11.2–33.0)*
Coastal Ostrobothnia, CO	2/34	5.9 (1.6–19.1)
Central Finland, CF	3/50	6.0 (2.1–16.2)
North Karelia, NK	6/46	13.0 (6.1–25.7)
Southwest Finland, SW	2/64	3.1 (0.9–10.7)*
Southeast Finland, SE	6/66	9.1 (4.2–18.4)
Age group		
Calf	16/171	9.4 (5.8–14.7)
Adult	15/171	8.8 (5.4–14.0)
Sex		
Female	13/150	8.7 (5.1–14.3)
Male	18/191	9.4 (6.0–14.4)
Unknown	0/1	0.0 (0.0–79.3)
Total	31/342	9.1 (6.5–12.6)

*CI* confidence interval

*Statistically significant difference (*p* = 0.005) and small effect size (*φ* = − 0.273) between districts

### Total Anti-HEV Antibodies in White-Tailed Deer and European Roe Deer

From the 70 studied white-tailed deer, we found one to be HEV seropositive (1.4%, CI 0.3–7.7%; Table 3). None of the 12 European roe deer sera were positive for anti-HEV antibodies (0.0%, CI 0.0–24.2%; Table 3).

**Table 3:** Total anti-HEV antibody prevalence in white-tailed deer and European roe deer in Finland

Variable	White-tailed deer		European roe deer	
	Number of samples (positive/total)	Seroprevalence % (95% CI)	Number of samples (positive/total)	Seroprevalence % (95% CI)
District				
Satakunta, S	1/20	5.0 (0.9–23.6)	0/6	0.0 (0.0–39.0)
Southwest Finland, SW	0/50	0.0 (0.0–7.1)	0/6	0.0 (0.0–39.0)
Age group				
Calf	0/35	0.0 (0.0–9.9)	0/3	0.0 (0.0–56.1)
Adult	1/35	2.9 (0.5–14.5)	0/9	0.0 (0.0–29.9)
Sex				
Female	0/26	0.0 (0.0–12.9)	0/4	0.0 (0.0–49.0)
Male	1/43	2.3 (0.4–12.1)	0/8	0.0 (0.0–32.4)
Unknown	0/1	0.0 (0.0–79.3)	–	–
Total	1/70	1.4 (0.3–7.7)	0/12	0.0 (0.0–24.2)

*CI* confidence interval

### HEV RNA in HEV Seropositive Samples

Due to the presence of slight PCR inhibition, two moose sera were tested also at 1:5 dilution. We did not detect HEV RNA in the serum samples from HEV seropositive moose (0/31; CI 0.0–11.0%) nor in the serum sample from HEV seropositive white-tailed deer (0/1; CI 0.0–79.3%).

### Evaluation of Sample Size

For estimating the seroprevalence for moose, our sample size was concluded to be more than adequate, for white-tailed deer nearly adequate, and for European roe deer inadequate.

## Discussion

We demonstrated for the first time the presence of anti-HEV antibodies in moose and in white-tailed deer in Finland. While this indicates exposure to the virus in these free-ranging wild cervids that are hunted for human consumption, no HEV RNA was found from the samples from HEV seropositive animals. Our study adds to the series of studies illustrating how testing samples from hunter-harvested game animals can be useful in investigating epidemiology of zoonotic infections (Jokelainen et al. 2010; Tonteri et al. 2016).

There have been studies on HEV prevalence in moose from only a few countries. Most of these studies were done in Sweden where HEV seroprevalence was 18.6% (Lin et al. 2015). Based on our results obtained with the same antibody assay kit, HEV also circulates in the moose population in Finland with a seroprevalence of 9.1%, approximately half of that reported from Sweden. However, Lin et al. (2015) detected no evidence of HEV in moose samples from the Northern parts of Sweden, while we found anti-HEV-antibodies also in moose from Northern Finland. This disparity may be partly due to the small number of samples (eight) from Northern Sweden (Lin et al. 2015) as distribution of moose (Lavsund et al. 2003) and geographical circumstances are similar in the Northern parts of Sweden and Finland. Reasons for differences in seroprevalence between Sweden and Finland remain unknown, but could include moose density, which is higher in Sweden than in Finland (Lavsund et al. 2003), and differences in the density of other animal reservoirs for HEV.

The geographical differences, including the statistically significant difference in HEV seroprevalences in moose between Southwest Finland and Northern Ostrobothnia districts, are noteworthy. The seroprevalence was over six times higher in Northern Ostrobothnia’s district, where moose density is high (Fig. 1), than in Southwest Finland. Lin et al. (2015) discussed previously that moose densities may have a potential positive correlation with HEV prevalence in the population. A similar correlation has been documented in wild boars (Michitaka et al. 2007; de Deus et al. 2008; Boadella et al. 2012; Larska et al. 2015). Our results, however, did not confirm such correlation in moose in Finland. This could be due to overall modest moose density differences between the districts with the maximal difference of less than two moose per 1000 ha. A positive correlation between higher HEV prevalence in wild boars and areal rurality has also been reported from Germany (Schielke et al. 2009) and Poland (Larska et al. 2015). In line with this, we recorded a significantly higher HEV seroprevalence in the North-East area where there are fewer urban areas than in the South-West area (SYKE 2013). It is noteworthy to notice, however, that most of Finland is classified as sparsely populated rural area (SYKE 2013). All districts in the North-East area also share a border with Russia. Unfortunately, data on moose density or HEV seroprevalence in Russia were unavailable. Therefore, the reasons behind the higher HEV seroprevalence in the North-East area are still unknown.

In Sweden, young adult moose had significantly higher HEV seroprevalence than moose calves, but no significant differences were observed when calves or young adults were compared with older adult moose (Lin et al. 2015). As we had only two age groups, calves and adults, the results by age group cannot be compared directly, and in our study, the HEV seroprevalences in the two age groups were similar. In most cervid studies, no difference has been detected in HEV seroprevalence between sexes (Lin et al. 2015; Zhang et al. 2015; Di Bartolo et al. 2017), which was also evident in our results.

Our study is the first to report a HEV seropositive white-tailed deer from Europe. White-tailed deer was introduced from the United States to few European countries in the second quarter of the twentieth century. Comparative HEV seroprevalence in wild white-tailed deer populations in the United States have been reported as 0.0% (Dong et al. 2011) and in Canada as 8.8% (Weger et al. 2017). Our estimate of HEV seroprevalence in wild white-tailed deer in Finland (1.4%) is in line with these estimates. The number of European roe deer samples was limited, and we found no HEV seropositive European roe deer. Similar results were also reported in European roe deer from the Netherlands (Rutjes et al. 2010) and Poland (Larska et al. 2015). Higher seroprevalences in European roe deer have been reported from Belgium, 3.0% (Thiry et al. 2017); Germany, 0.0–6.8% (Neumann et al. 2016; Anheyer-Behmenburg et al. 2017); Sweden, 6.7% (Roth et al. 2016); and Spain, 10.4% (Boadella et al. 2010). Overall, the HEV seroprevalence in deer in Finland appears to be lower than those reported from other European countries, including Germany (Neumann et al. 2016), Sweden (Roth et al. 2016), Spain (Boadella et al. 2010; Kukielka et al. 2016), and Italy (Di Bartolo et al. 2017), although the results are not directly comparable due to different sampling strategies and serology methods.

In this study, we were unable to identify which HEV genotypes circulate in moose and in deer in Finland as we detected no viremia in the HEV seropositive animals. Finding antibodies against HEV but not HEV RNA from cervid sera has also been documented in other studies (Neumann et al. 2016; Roth et al. 2016; Weger et al. 2017). Multiple reasons could explain the lack of detectable HEV RNA, including low HEV RNA titre, quality of the samples, and lack of acute infections in seropositive animals. In our study, the upper limit of the 95% CI of HEV RNA prevalence was under 11.0%, which is in line with the results from Sweden where 4.3% of all moose had both anti-HEV antibodies and HEV RNA (Lin et al. 2015). So far, HEV from moose has been genotyped only in Sweden, although HEV RNA has also been found from one moose from Lithuania (Spancerniene et al. 2018). As the HEV genotype in moose is potentially new and with unknown zoonotic potential (Lin et al. 2014, 2015; Roth et al. 2016), further studies are needed in the regions where moose are commonly hunted for human consumption.

As only one deer sample was screened for HEV RNA in our study, the chance of finding HEV RNA for genotyping from deer was limited. Since HEV-3 circulates in the Finnish pig population (Kantala et al. 2013, 2015) and only zoonotic HEV-3 has been found from deer in Europe, it can be speculated that zoonotic HEV-3 could cause HEV infections in white-tailed deer in Finland as well. Using protective gloves during disembowelling hunted animals has been previously connected to lower HEV seroprevalence in hunters (Schielke et al. 2015). Therefore, good hygiene during handling hunted deer and harvesting the meat is advisable. Venison should not be eaten raw but thoroughly cooked.

In conclusion, our results indicate that HEV circulates in European moose population in Finland and HEV infections may also occur in white-tailed deer population. It remains to be shown whether HEV infections occur in the local population of European roe deer. It seems that the HEV seroprevalences in cervids in Finland were not as high as in other European countries, including Germany, Italy, Spain, and Sweden. Therefore, it is possible that the potential risk for zoonotic HEV transmitted by hunting cervids and eating their meat is not as high as in these countries. However, the samples used in this study were collected several years ago and the epidemiological situation may have already changed. Regardless, our study provides the first estimate of the prevalence and establishes a baseline that can be used for comparison in future studies. Further studies are needed to demonstrate which HEV genotypes the cervids in Finland carry and to evaluate the importance of these findings to food safety.
